# GHG mitigation in Agriculture, Forestry and Other Land Use (AFOLU) sector in Thailand

**DOI:** 10.1186/s13021-019-0119-7

**Published:** 2019-04-23

**Authors:** Bijay Bahadur Pradhan, Achiraya Chaichaloempreecha, Bundit Limmeechokchai

**Affiliations:** Sirindhorn International Institute of Technology, Thammasat University, Pathumthani, Thailand

**Keywords:** Agriculture Forestry and Other Land Use (AFOLU), Greenhouse gas emission mitigation, AFOLUB model, Thailand

## Abstract

**Background:**

The Agriculture, Forestry and Other Land Use (AFOLU) sector is responsible for almost a quarter of the global Greenhouse gases (GHG) emissions. The emissions associated with AFOLU activities are projected to increase in the future. The agriculture sector in Thailand accounted for 21.9% of the country’s net GHG emissions in 2013. This study aims to estimate the GHG emissions in the AFOLU sector and mitigation potential at various carbon prices during 2015–2050. This study uses an AFOLU bottom-up (AFOLUB) model to estimate GHG emissions in a business-as-usual (BAU) scenario, and then identifies no-regret options, i.e. countermeasures that are cost-effective without any additional costs. In addition, the study also identifies countermeasure options and mitigation potential at various carbon prices.

**Results:**

Results show that emissions from the agriculture sector in the BAU will increase from 45.3 MtCO_2_eq in 2015 to 63.6 MtCO_2_eq in 2050, whereas net emission from the AFOLU will be 8.3 MtCO_2_eq in 2015 and 24.6 MtCO_2_eq in 2050. No-regret options would reduce emissions by 6.1 and 6.8 MtCO_2_eq in 2030 and 2050, respectively. The carbon price above $10 per tCO_2_eq will not be effective to achieve significant additional mitigation/sequestration.

**Conclusions:**

In 2050, no-regret options could reduce total AFOLU emissions by 27.5%. Increasing carbon price above $10/tCO_2_eq does not increase the mitigation potential significantly. Net sequestration (i.e., higher carbon sequestration than GHG emissions) in AFOLU sector would be possible with the carbon price. In 2050, net sequestration would be 1.2 MtCO_2_eq at carbon price of $5 per tCO_2_eq, 21.4 at $10 per tCO_2_eq and 26.8MtCO_2_eq at $500 per tCO_2_eq.

## Background

The Agriculture, Forestry and Other Land Use (AFOLU) is a term that is used in 2006 Intergovernmental Panel on Climate Change (IPCC) Guidelines which describes the anthropogenic GHG emissions from two distinct sectors: Agriculture and LULUCF (Land Use, Land Use Change and Forestry), which were previously treated separately. AFOLU sector is one of the contributors of greenhouse gas (GHG) emissions globally, producing about one-fourth of global GHG emissions [1]. Developing countries are accountable for the majority of GHG emissions. Asia has the highest share in the global AFOLU emission. The increasing emissions are mainly due to deforestation and agricultural emissions. The contribution of developing countries in AFOLU related emissions is expected to increase significantly in future due to projected increase in food production and land conversions.

In the context of climate change, the agriculture sector is crucial because it should not be opposed to United Nations Framework Convention on Climate Change (UNFCCC) objective of a stable food supply, as food is a must for human survival. Therefore, mitigation policies in the agriculture sector should reflect a win–win strategy [2]. Thailand submitted its Intended Nationally Determined Contributions (INDC) to the UNFCCC, but the INDC targets are mainly focused on energy related emission reduction targets and not on the AFOLU sector. However, Thailand has stated in its INDC an intent to maintain a forest area of 40% of the total land area [3] but there is no quantifiable emission reduction target in case of agriculture sector. The Office of Natural Resources and Environmental Policy and Planning (ONEP) has mentioned some of the mitigation measures in the land-use sector, which include reforestation, forest conservation, maintenance of biological richness in marine and coastal resources, rehabilitation of watershed areas and tree plantation in abandoned land. Measures in the agriculture sector include reducing agricultural open-burning, and biogas installations [2].

The Paris Agreement invites all countries to include land-based mitigation options and to take action on REDD+ (Reducing Emissions from Deforestation and forest Degradation plus conservation of forest carbon stocks, sustainable management of forests, and enhancement of forest carbon stocks) to increase mitigation from Land use sector. The CO_2_ in the atmosphere can be stored as carbon in terrestrial vegetation and soils. Under UNFCCC, any mechanisms or activities that removes the atmospheric CO_2_ is termed as sink. LULUCF is the second highest contributor to GHG emissions after fossil fuel combustion. The activities in LULUCF sector can contribute in climate change mitigation through accumulation of atmospheric CO_2_ in the form of carbon stocks in land. Forests can play an important role in mitigation of climate change either through reducing the loss of net carbon stocks or by increasing the average carbon stocks in long term. Federici et al. [4] estimated the global and regional trends in net emissions and removals from forest land (including net forest conversion and remaining forest) using the data of Forest Resources Assessment 2015. The study found that the net forest conversion during the period 2011–2015 has become lower than during the period 2001–2010. Grassi et al. [5] quantified global LULUCF net GHG flux for altogether 195 UNFCCC countries in four different scenarios. The study concluded that the LULUCF sector, which contributed to the emission of 1.3 ± 1.1 GtCO_2_eq/year during 1990–2010, can be a net sink of carbon by 2030 with the implementation of the countries’ INDC. Forest coverage varies widely across the world. Some industrialized nations like Japan, Korea, Finland, Sweden and Malaysia have more than 60% of its land as forests. Thailand’s forest area was only 33% in 2015. Thai government’s target to maintain 40% land as forest area is still quite low when compared to the existing forest coverage in Southeast Asian countries except Singapore and Philippines [6, 7]. More ambitious forest targets can help to sequester more carbon dioxide and offset GHG emissions from other sectors.

Mitigation measures in the AFOLU sector include sequestration as well as reduction in the emissions from livestock and agricultural processes. There are two ways to achieve mitigation in the AFOLU sector i.e. through supply-side measures and demand-side measures. Supply side measures include reducing emissions through livestock management, land management and land-use change, and increasing sequestration from afforestation. Demand-side measures include changes in eating habits and reducing food wastes; however, quantitative measures for demand-side measures are more uncertain [1]. A study by the United States Environmental Protection Agency (USEPA) has quantified non-CO_2_ emissions in baseline and analyzed mitigation potential and marginal abatement costs (MACs) of various countermeasures by sector and regions [8]. Graus et al. [9] carried out a similar study for the agriculture sector and estimated MACs for the year 2020 and 2050. Many models that analyzed the economic costs of GHG abatement, carbon prices are used as proxies to represent level of efforts in mitigation policies [10]. Smith et al. [1] reported that in the AFOLU sector, mitigation possible from supply-side measures with carbon prices up to 100 United States dollars (US$) per tCO_2_eq is in the range of 7.18 to 10.60 GtCO_2_eq/year in 2030, about one-third of which can be achieved at below $20 per tCO_2_eq. Nabuurs et al. [11] stated that the economics of carbon sequestration projects in developing countries is in the range of $0.5 to $7.0 per tCO_2_eq, whereas in the case of developed countries the costs are in the range of $1.4 to $22 per tCO_2_eq, based on costs compiled for different regions by Cacho et al. [12] and Richards, Stokes [13]. Graham et al. [14] estimated the cost of carbon emission reduction through various REDD+ strategies in Southeast Asia; the cost of reducing emissions is in the range of $9 to $75 per ton of carbon (tC) emission avoided.

To the author’s knowledge, there are very few studies that have quantified the mitigation possibilities from various countermeasure options in the case of Thailand. The Center for Applied Economic Research (CAER) in Thailand identified some of the mitigation options in the agriculture sector based on interviews with the experts in the related field [15]. The identified options include improved feed quality for livestock in enteric fermentation, alternative wetting and drying in rice cultivation, anaerobic digesters replacing uncovered lagoons in manure management, appropriate fertilizer application for site-specific nutrient management in managed soils, and reducing or preventing agro-residue burning in the field. In addition, the study also quantified mitigation potential in different adoption rates (i.e. low, medium, high). However, the study did not consider the cost required during implementation of such countermeasures [15].

The objective of this study is to assess the GHG emissions from the AFOLU sector in the Business-as-usual (BAU) scenario during 2015–2050 by using the AFOLUB model. In addition, it also identifies the optimal (i.e., profit maximizing) set of GHG mitigation/sequestration options from the sector at wide ranging values of the carbon price and estimates their corresponding GHG mitigation potential during the period.

## Methods

**Fig. 1:**
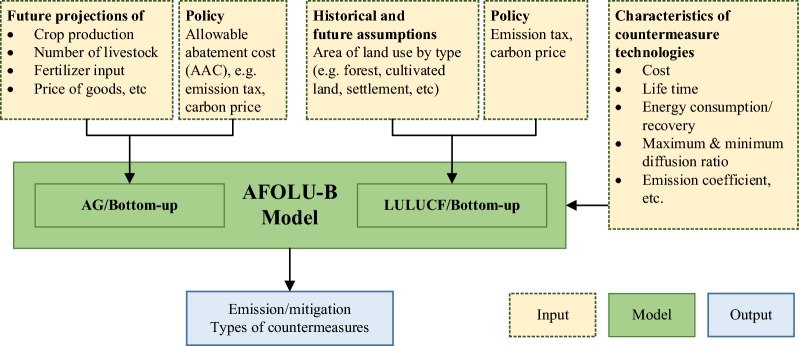
Framework of AFOLU-B model

The study uses the Agriculture, Forestry and Other Land Use Bottom-up (AFOLUB) model for the analysis. The AFOLUB model was developed by the joint effort of Kyoto University, Japan, and the National Institute for Environmental Studies (NIES), Japan. Figure 1 presents the framework of the AFOLUB model. This model is applicable for both national and regional level analysis. The GHG emissions considered in the model are CO_2_, N_2_O and CH_4_. The AFOLUB model is a bottom-up model for the analysis of GHG emission and mitigation. Several studies have used the model to study GHG emission and identified cost-effective mitigation options and potential in the AFOLU sector under different abatement costs at national level in the case of Bangladesh [16], Nepal [17], Vietnam [18], Indonesia and Malaysia [19, 20]. A general equilibrium analysis of Indonesia in the land-use sector was also carried out using the AFOLUB model combined with AIM/CGE model [21].

The AFOLUB model requires three types of inputs given exogenously into the model. The first input is the future projection of agricultural production and area of land use change in the baseline case. The second input requires detailed information of countermeasure options that includes cost and mitigation impact of each option. The countermeasure option refers to the emission mitigation or abatement measures. The last input includes policy scenarios that includes emission tax which represents the willingness to achieve GHG reduction. The AFOLUB model consists of two modules. The first module includes analysis for the agricultural sector, termed the “AG/Bottom-up model”, and the second for Land-use, land-use change and forestry (LULUCF) sector, which is termed the “LULUCF/Bottom-up model”. These two models used two different objective functions in order to determine the countermeasure options which are discussed in the subsequent sections.

The AG/Bottom-up model takes into account non-CO_2_ gases i.e., CH_4_ and N_2_O. The model determines the combination of selected countermeasures in order to maximize the profit. The countermeasures for various sources of emissions such as enteric fermentation, rice cultivation, manure management, and managed soils from agricultural activities are determined by the model. The profit is calculated as the sum of revenues from agricultural production and energy recovery minus the sum of production and mitigation costs. The production cost is the sum of initial cost, labor cost, energy cost and cost of carbon emissions. The revenue from energy recovery in manure management by the use of biogas production technology refers to savings in cooking cost with the use of biogas. For other technologies in agriculture sector, revenues are not considered. This model considers only livestock and does not include fishery and aquatic animal farming. In this model, the cost of countermeasure is assumed to remain the same throughout the study period. The chances of decrease in countermeasure costs in future are not considered.

**Fig. 2:**
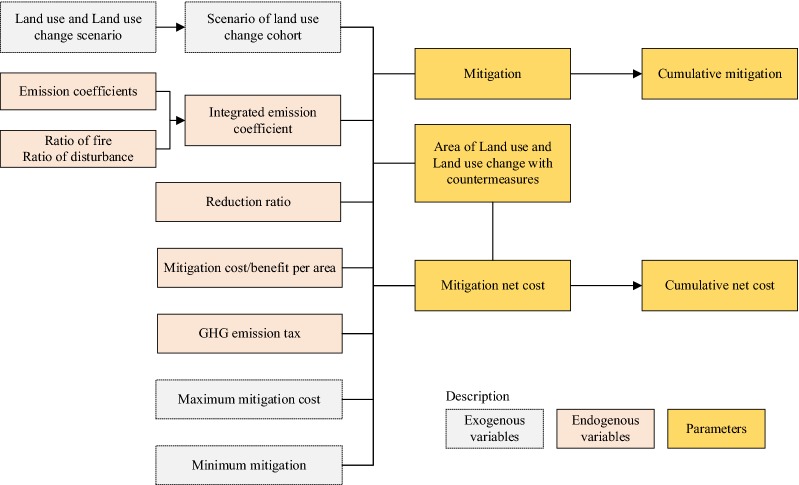
Framework of Land Use model

The LU-Bottom-up model calculates emissions and sinks from land-use changes. It also accounts for the emissions from forest fires, other natural disturbance of forest and peat drainage. The framework of Land Use model is shown in Fig. 2. The change in land use pattern is given exogenously in the model. Land use is divided into five categories i.e. forest, grassland, cultivated land, settlement and other land. Other land includes barren land and non-cultivated agricultural lands. The model estimates the GHG emission and sequestration from LULUCF using carbon stock difference equations of the 2006 IPPC guidelines. Mitigation is calculated as the product of area of land (where mitigation is applied) and emission coefficient. The model also considers the change in carbon stock over time as a result of change in carbon sequestration rate with the age of the forest. Unlike AG/Bottom-up model that determines countermeasure options by maximizing the profit, the LU-Bottom-up model determines the countermeasures by maximizing mitigation potential under cost constraints. It should be noted that the model does not account for the cost of forest clearing, land preparation and revenue from wood production. The model also requires several constraints that include total annual area, availability of area to apply countermeasures, and no overlapping of countermeasures. Overlapping of countermeasures is restrained in the model. It is also assumed that once the land is converted to another category, the land use is continued for several decades. For more details of the AFOLUB model and formulas used for calculations, refer to Hasegawa, Matsuoka [19].

In this study, the data on agriculture and forestry in Thailand are required. In the agriculture sector, the data for the base year as well for future years, i.e. the study period, are required. The data required are production, yields, prices of commodities, and fertilizer inputs in the case of cultivated crops. In the case of livestock, the data required are number of livestock and prices of livestock. Unlike agriculture sector, in the case of land-use, the model requires historical land-use patterns as well as future land-use patterns. The costs and mitigation potential of the countermeasures considered in the analysis are also required. Since the AFOLUB model uses emission tax rather than revenues from carbon abatement, the carbon tax is used as a proxy to carbon price to measure mitigation potential.

It should be noted here that the model requires only the extra cost associated with the application of countermeasures as an input to the model. The extra cost includes the additional cost which is equal to the difference between cost of production with countermeasure and cost in the base case. This extra cost includes difference in the investment cost, O&M cost, additional wage cost and cost of emission tax (if applicable). For example, if the manure management in the base case considered daily spread of manure, and the countermeasure considered biogas production using anaerobic digester. Then the extra wage cost is the difference in the wage cost of daily spread of manure and the wage cost in operating the biogas digester.

### Overview of AFOLU sector

Agricultural land in Thailand has not changed significantly during 2003–2015 with the area covering about 47% of the total land area. Forest area in Thailand has changed significantly during 1987–2015. Deforestation occurred during 1985–1998 with forest area reducing from 29.4 to 25.3%. However, afforestation led the forest area to increase to 33.2% in 2000. The forest area decreased to 30.9% in 2006 [22] and then increased to 33.0% in 2015 [23].

**Fig. 3:**
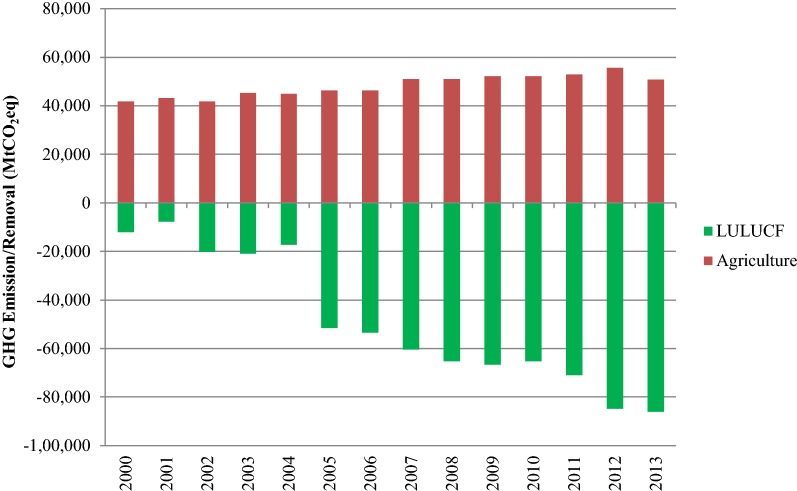
Emission/removal trend in AFOLU sector during 2000–2013

Total GHG emission from the agriculture sector increased from 41.9 MtCO_2_eq in 2000 to 46.2 in 2005 and 50.9 MtCO_2_eq in 2013 (see Fig. 3) with a compound annual growth rate 1.51% per year during 2000–2013. The emission from agriculture sector in 2013 accounted for 21.9% of the net emission in the country. Most of the emission in agriculture sector comes from rice cultivation (54.72%) followed by agricultural soils (22.95%), enteric fermentation (11.79%), manure management (6.95%), and field burning of agricultural residues (3.59%).

The LULUCF sector showed a trend of increased net removal. LULUCF activities in Thailand contributed to the net removal from atmosphere since 2000. From 2005, the net removal of LULUCF sector included rubber plantations in the calculation. Thus, the results in Change in Forest and Other Woody Biomass Stock showed a tremendous increase of CO_2_ removal. In Thailand’s Second National Communication, rubber plantation has not been accounted as forest [24]. The LULUCF sector contributed to the net removal of 11.9 MtCO_2_eq in 2000, 51.5 MtCO_2_eq in 2005 and 86.1 MtCO_2_eq in 2013 (seven-fold higher compared with 2000) with a compound annual growth rate 6.62% per year during 2005–2013 [25].

### Input data and assumptions

#### Crops

**Fig. 4:**
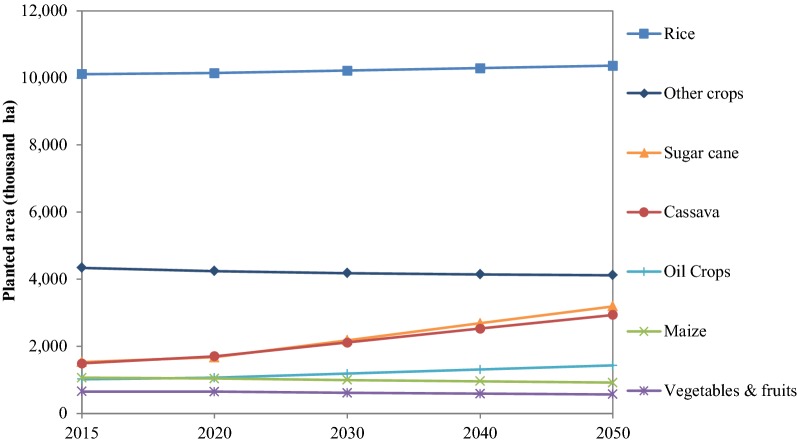
Estimated crop cultivated area during 2015–2050

Data in the base year on crop production and cultivate area are based on annual reports published by the Office of Agricultural Economics (OAE) in Thailand [26–29]. The information in the baseline scenario for crop-cultivated areas is based on historical growth rates. In this study, rice cultivated area is assumed to grow at an annual growth rate of 0.07%, while maize cultivated area is assumed to decline at 0.41% annually (see Fig. 4). Both are based on compound annual growth rate (CAGR) during 2005–2015. The cultivated area for cassava, oil crops and sugarcane in the future years is estimated using linear function based on time series analysis from 2005 to 2015 data. Similarly, for vegetables and other crops, the projection is based on time series analysis using logarithmic function. The crop yield data and all the prices of commodities are also based on annual reports published by the OAE [26–29]. The effect of climate change and socio-economic changes on the crop yield in future has not been considered in this study and estimated by time series analysis based on historical data (2005–2015). These are some of the limitations in the data forecast in this study.

#### Livestock

The projected livestock population is shown in Table 1. In this study, several approaches are used for livestock projection. Dairy cattle are assumed to grow at an annual growth rate of 0.62% during 2015–2050 based on historical CAGR during 2005–2015 [26–29]. Beef cattle and buffaloes showed a decline in numbers during 2005–2015. However, in this study populations of both livestock during 2015–2050 are assumed to remain the same as in 2015. Swine and sheep populations are assumed to change annually at 1.9% and − 0.27%, respectively, based on historical growth/decline patterns. Goats, chicken and duck populations are assumed to grow based on time series analysis using 2005–2015 data.

**Table 1:** Estimated livestock population during 2015–2050 (thousand heads)

Livestock type	2015	2020	2030	2040	2050
Cattle (dairy)	510	526	559	595	633
Cattle (beef)	4407	4407	4407	4407	4407
Buffaloes	888	888	888	888	888
Sheep	49	49	48	46	45
Goats	540	587	756	924	1092
Horses	4	4	4	4	4
Swine	9887	10,873	13,151	15,906	19,238
Chickens	418,331	490,288	677,284	864,280	1,051,275
Ducks	28,762	36,782	46,282	55,782	65,283

#### Projection of land-use data

**Fig. 5:**
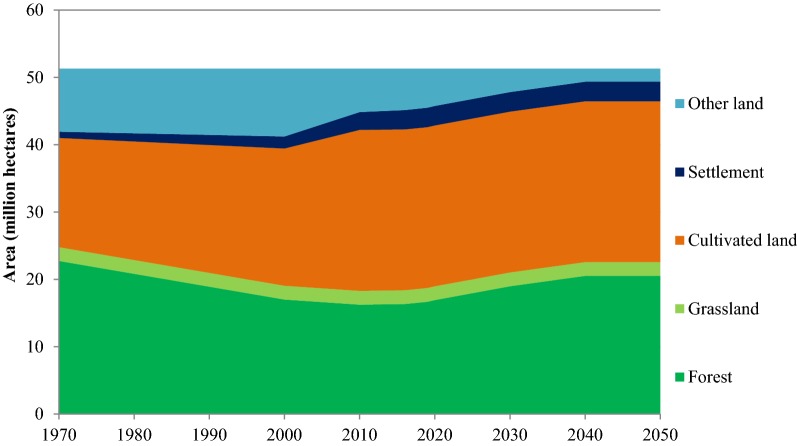
Estimated land-use change patterns in Thailand during 2015–2050

Land-use patterns for Thailand have been taken from various sources for different years [22]. Thailand has set the target to increase the forest area to 40% of the total land area [3], but in 2015 only 33.0% of total land area is forest [23]. Therefore, in this study, forest area is assumed to gradually increase to 40% by 2030 and remain at 40% thereafter until 2050 (see Fig. 5). Protected forests are assumed to be 20% of the total land area, i.e. 60.6% of the total forests are protected forests while the remaining 39.4% are production forests [22]. Rubber plantation in this study is not considered as forest land, and has been included in agricultural land following Thailand’s Second National Communication submitted to the UNFCCC [24]. Grassland areas are assumed to remain constant. Settlement areas are projected based on the population and it is assumed that the settlement area per person would remain the same as in year 2016 until 2030. During 2030–2050 the settlement area is assumed to be constant as the population is expected to decrease. Population projection is based on projection reported by Office of the National Economic and Social Development Board (NESDB). Thailand’s population growth rate was 0.5% during 2010–2017, relatively lower than in the past years. The assumptions suggest fertility rate to become stable and then decrease in future. The fertility rate is assumed to become lower until 2030 and then decrease. The aged population in Thailand’s is expected to become one and half times than of the youth population by 2030 [30]. Other land (which includes barren lands and non-cultivated agricultural land) is calculated as the difference between total area and the sum of forest, grassland, settlement and cultivated land. This study does not consider the shift in land use pattern due to changes in economic activities.

#### Emission sources

In order to estimate the emissions from the agriculture and LULUCF sectors, the AFOLUB model classified the emission sources based on the 2006 IPCC Guidelines for National Greenhouse Gas Inventories (2006 IPCC Guidelines) [31]. The emissions from the agriculture sector are classified into four categories: enteric fermentation, manure management, rice cultivation and managed soils. In the LULUCF sector, the emission/sequestration is classified into three categories: changes in carbon stock and other woody biomass, forest and grassland conversion, and emission and removals from soils. The equations to estimate emissions from different sources and emission factors are also based on the 2006 IPCC Guidelines.

#### Mitigation countermeasures

The data on countermeasure options are obtained from various national sources as well as international sources when national data are not available. Tables 2 and 3 present countermeasures in the agriculture and LULUCF sectors considered in this study, respectively. All costs are given in 2010 US$. The study has considered only the supply-side measures and ignores the demand-side mitigation measures in the analysis.

**Table 2:** Countermeasures in the agriculture sector

Emission sources	Mitigation options	Unit	Cost/unit (in 2010 US$)	Mitigation (tCO_2_eq/unit/year)	References
Enteric fermentation	Improved feeding (replacing roughage with concentrates)	Head	− 21.2	0.45	[18, 19]
	High genetic merit	Head	0	0.32	[32–34]
Manure management	Dome digester	Head^a^	44	0.62	[35]
	Daily spread of manure	Head	2.2	0.33	[35]
Rice cultivation	Midseason drainage	Hectare	0	0.36	[9, 32]
	Incorporation of off-season rice straw	Hectare	0	0.45	[9, 32]
	Replace urea with ammonium sulphate	Hectare	1.5	0.12	[9, 32]
Managed soils	High-efficiency fertilizer application	Hectare	32	0.65	[9]
	Slow-release fertilizer application	Hectare	2150	0.76	[35]
	Tillage and residue management	Hectare	5	0.08	[36]

^a^The dome digester cost has been converted into cost per head by dividing the cost by number of cows/buffaloes

**Table 3:** Counter measures in the LULUCF sector

Mitigation options	Cost (US$/ha/year)	Mitigation (tCO_2_eq/ha/year)
Sustainable management of production forest areas^a^	15.4	11.3
Conservation of existing protection forests^b^	23.0	11.1
Reforestation^b^	58.1	23.6
Planting long-rotation large timber trees^a^	9.3	19.6
Growing long-rotation non-timber product forest^a^	7.0	14.6
Reduced impact logging^b^	27.8	5.1

Sources: ^a^Hoa et al. [18], ^b^Graham et al. [14]

#### Scenario descriptions

The study has considered nine scenarios for the analysis. The scenarios include one BAU scenario, one No Climate Policy (NCP) scenario and seven carbon price scenarios.

##### Business-as-usual scenario

In the BAU scenario, no mitigation technologies/measures are considered, i.e., there are no countermeasures applied for GHG mitigation during 2015–2050. In addition, no carbon policy options are considered, i.e. no carbon price is considered. The assumptions of livestock, crop production and land-use pattern in the future years are the same as presented in aforementioned sub-sections. The summary of the projected values considered in the BAU are presented in the Table 4.

**Table 4:** Summary of the projected parameters in the BAU. Source: For agriculture [26–29], For land use [22]

Indicators	Assumptions	Equation*	R^2^
Agriculture			
Crop area (thousand hectares)	Time series analysis using 2005–2015 data		
Rice	Historical growth rate (0.07% p.a.)	–	–
Cassava	Using linear function	y = 41.0x + 1049	0.76
Maize	Historical growth rate (0.41% p.a.)	–	–
Vegetables	Using logarithmic function	y = − 75.6ln(x) + 860	0.90
Oil crops	Using linear function	y = 12.3x + 865	0.76
Sugarcane	Using linear function	y = 50.7x + 861	0.76
Other crops	Using logarithmic function	y = − 166ln(x) + 4562	0.64
Livestock (thousand heads)	Time series analysis using 2005–2015 data		
Cattle (dairy)	Historical growth rate (0.62% p.a.^a^)	–	–
Cattle_(other)	Constant during 2015–2050	–	–
Buffaloes	Constant during 2015–2050	–	–
Swines	Historical growth rate (1.9% p.a.^a^)	–	–
Goats	Liner regression	y = 16.8x + 318	0.71
Sheep	Historical growth rate (− 0.27% p.a.^a^)	–	–
Horses	Constant during 2015–2050	–	–
Duck	Liner regression	y = 950x + 21,582	0.45
Chicken	Liner regression	y = 18,699x + 191,094	0.79
Land use			
Forest	Government target to achieve 40% [6]	–	–
Grassland	Constant with 2015	–	–
Agricultural	Constant with 2015	–	–
Settlement	Settlement area per capita constant Population projection based on government [30]	–	–

*p.a.* per annum

^a^x in the equation is independent variable

##### No Climate Policy scenario

The NCP scenario is the same as the BAU scenario except that it considers the countermeasure options discussed in aforementioned sub-section in Tables 2 and 3. Similar to the BAU scenario, the NCP scenario does not consider any carbon prices. The selection is based on the cost and mitigation of the countermeasures. This scenario is considered as it helps to identify no-regret options, i.e. options that are cost-effective without any carbon prices.

##### Carbon price scenarios

The carbon price scenarios are the same as in the NCP scenario except that they consider carbon prices. In this study, seven scenarios of carbon prices are considered, namely $5, $10, $25, $50, $100, $300 and $500 per tCO_2_eq. It should be noted that all the prices are in 2010 US$.

## Results and discussion

### GHG emissions in the BAU scenario

**Fig. 6:**
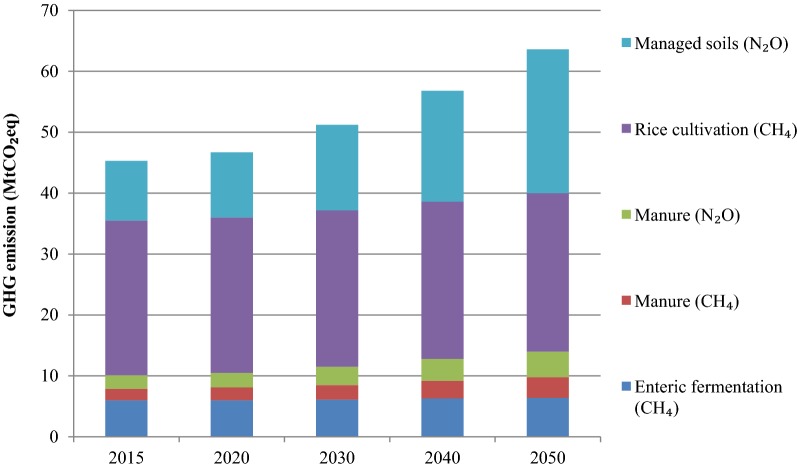
Emissions from the agriculture sector during 2015–2050

The GHG emission from the agriculture sector in 2015 was estimated to be 45.3 MtCO_2_eq. The emissions will reach 51.2 MtCO_2_eq in 2030 and 63.6 MtCO_2_eq in 2050 (see Fig. 6). The methane emissions from rice cultivation accounts for the highest GHG emissions in the agriculture sector in 2015 and thereafter during 2015–2050. In 2015, the emissions from rice cultivation were 25.4 MtCO_2_eq. There will be no significant changes in methane emission from rice cultivation and the emissions would increase only slightly to 26.0 MtCO_2_eq in 2050. The emissions from enteric fermentation will increase from 6.0 MtCO_2_eq in 2015 to 6.4 MtCO_2_eq in 2050. The methane (CH_4_) emissions from manure management would increase by 80% during 2015–2050, from 1.9 MtCO_2_eq in 2015 to 3.4 MtCO_2_eq in 2050. During the same period, N_2_O emissions from manure management is estimated to nearly double from 2.2 MtCO_2_eq in 2015 to 4.2 MtCO_2_eq in 2050. The emissions from managed soils would increase from 9.8 MtCO_2_eq in 2015 to 23.6 MtCO_2_eq in 2050, an increase of 1.4 times from the 2015 level.

In 2015, rice cultivation had the highest share (56.1%) in GHG emissions in the agriculture sector. In 2015, the N_2_O emissions from agricultural soils were the second major contributor of GHG emissions in terms of CO_2_ equivalent with the share of 21.6%, followed by CH_4_ emissions from enteric fermentation (13.2%), N_2_O emissions from manure (4.9%) and CH_4_ emissions from manure (4.2%). During 2015–2050, rice cultivation would remain the major contributor to GHG emissions in the agriculture sector in the BAU scenario. However, the share of rice cultivation in GHG emission in 2050 would decrease to 40.9%. In 2050, the agricultural soils would remain the second biggest contributor of GHG emission with the share of 37.1%. The share of enteric fermentation would be 10.1% while that of CH_4_ and N_2_O emissions from manure would be 5.3% and 6.6%, respectively.

**Fig. 7:**
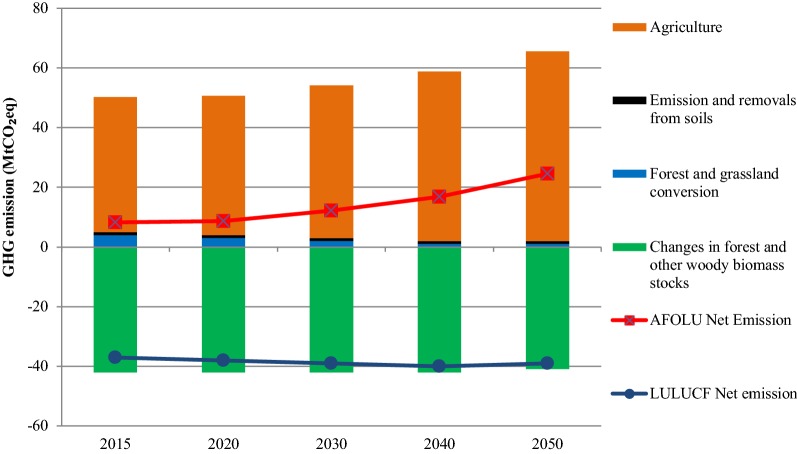
Emissions from the AFOLU sector during 2015–2050

Figure 7 presents the emission from the agriculture sector, emission as well as sequestration from the LULUCF sector, net sequestration from LULUCF sector and net emission from the AFOLU sector. In LULUCF, forest sinks, i.e. changes in forest biomass and other woody biomass stocks, sequestered 42.0 MtCO_2_eq in 2015. It should be noted here that carbon sequestration from rubber plantation has not been accounted in this analysis, therefore, there is high variation in the sequestration results in this study and Thailand’s Third National Communication submitted to the UNFCCC [25]. In this analysis, rubber plantation is not considered as the forest data provided by Land Development Department of the government does not account rubber plantation as forest. During 2015–2050, the sink capacity would slightly decrease to 41.0 MtCO_2_eq in 2050. The emissions from forest and grassland conversion will decrease from 4.0 MtCO_2_eq in 2015 to 1.0 MtCO_2_eq in 2040 and will remain unchanged afterwards. The emission and removals from soil will contribute to about one MtCO_2_eq of GHG emission during 2015–2050. There would be net sequestration from the LULUCF sector, i.e., emission removal from sink would be greater than the emissions in the LULUCF. Net sequestration from LULUCF would increase from 37.0 MtCO_2_eq in 2015 to 40.0 MtCO_2_eq in 2040. The level of sequestration would be 39.0 MtCO_2_eq in 2050. Overall, in the AFOLU sector there will be net emission throughout the study period 2015–2050. Although there is no significant change in the emission and sequestration pattern in the LULUCF sector, the net emission from the AFOLU sector will increase during 2015–2050 due to the increase of emissions from the agriculture sector. The net emissions will increase from 8.3 MtCO_2_eq in 2015 to 12.2 MtCO_2_eq in 2030 and 24.6 MtCO_2_eq in 2050.

The emission from the AFOLU sector is dependent on the crop and livestock production and land-use change pattern. Crop and livestock production are also affected by climate change and socio-economic conditions of a country. A study by CAER has projected crop production in various climate and socio-economic change scenarios. The production in different scenarios in future varied from the business-as-usual scenario, although the variation was not to a great extent [15]. The consideration of such changes will change the emission forecast in the AFOLU sector. This study has considered only one scenario for agricultural demand and land-use change in the analysis. This study intends to give an insight on the estimation of the emission in the AFOLU sector and possible mitigation potential at various carbon prices, therefore, the effect of such changes has not been focused in the study.

### GHG Mitigation potential in various scenarios

This section discusses the emission mitigation and carbon sequestration potential in the agriculture and LULUCF sectors of various countermeasures under different scenarios discussed earlier. In addition, cumulative GHG emissions during 2015–2050 in various scenarios are also presented in this section.

### Mitigation from the agriculture sector

**Fig. 8:**
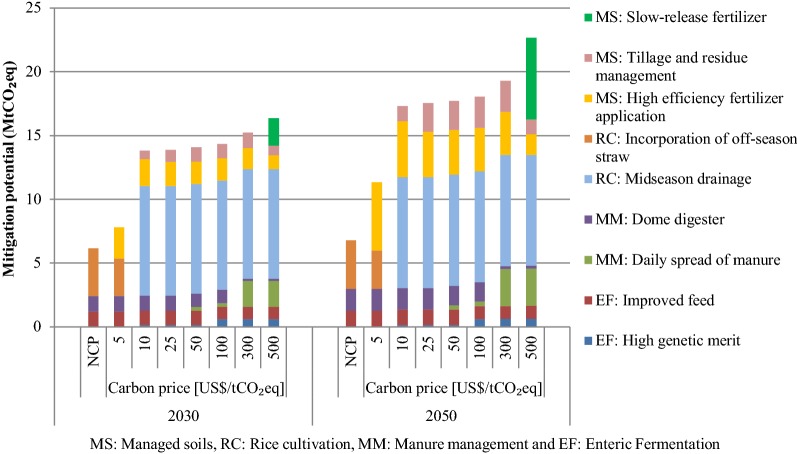
GHG mitigation potential in the agriculture sector in 2030 and 2050 in various scenarios

GHG emission reduction options in agriculture can be categorized into enteric fermentation, manure management, rice cultivation and agricultural soils. Figure 8 shows the mitigation potential of various countermeasures in the agriculture sector in 2030 and 2050. In the NCP scenario, the cost-effective mitigation options in 2030 include improved feed in enteric fermentation, dome digesters in manure management, and incorporation of off-season straw in rice cultivation. In 2030, the total mitigation potential in the agriculture sector in the NCP scenario will be 6.1 MtCO_2_eq. At a carbon price of $5/tCO_2_eq, the countermeasures include high-efficiency fertilizer application in managed soils, in addition to countermeasures in the NCP scenario. In this scenario, the mitigation potential of all countermeasures will be 7.9 MtCO_2_eq in 2030. At $10/tCO_2_eq, the countermeasures that are cost-effective include high genetic merit and improved feed in enteric fermentation, dome digesters in manure management, incorporation of off-season straw in rice cultivation; and high efficiency fertilizer application and tillage and residue management in managed soils. There are no changes in selected countermeasures at $25/tCO_2_eq when compared to $10/tCO_2_eq. In 2030, the mitigation potential at carbon prices of $10/tCO_2_eq and $25/tCO_2_eq would be 13.8 and 13.9 MtCO_2_eq, respectively. At carbon prices of $50, $100 and $300 per tCO_2_eq, daily spread of manure in manure management is an additional countermeasure while the remaining options are the same as in $10 and $25 per tCO_2_eq. The mitigation potential at $50, $100 and $300 per tCO_2_eq is 14.1, 14.3 and 15.2 MtCO_2_eq, respectively. At the higher carbon price of $500/tCO_2_eq, the mitigation potential will be 16.4 MtCO_2_eq. All the countermeasures are the same as in $300 per tCO_2_eq, except the use of slow-release fertilizer in managed soils, which is an additional countermeasure option at this carbon price.

In 2050, the selected countermeasures in all scenarios are the same as in the year 2030. In the NCP and $5/tCO_2_eq scenarios, mitigation potential would be 6.8 and 11.3 MtCO_2_eq, respectively. At $10, $25 and $50 per tCO_2_eq, the mitigation would be 17.3, 17.5 and 17.7 MtCO_2_eq, respectively. Likewise, the mitigation at $100, $300 and $500 per tCO_2_eq would be 18.0, 19.3 and 22.7 MtCO_2_eq, respectively.

Similar studies for other countries have also reported improved feed, dome digesters and incorporation of off-season straw as no-regret options, i.e. cost-effective without carbon price. In the case of Bangladesh, Indonesia, Nepal and Vietnam, dome digesters in manure management and improved feed in enteric fermentation are reported as no-regret options. Other studies for the same countries have reported high genetic merit in enteric fermentation as a no-regret option; however, in this study it is found to be cost-effective at $10/tCO_2_eq and higher carbon prices. Similar to the case of Nepal, incorporation of off-season straw in rice cultivation is also a no-regret option. The studies for Vietnam, Indonesia and Malaysia found mid-season drainage in rice cultivation to be a no-regret option; however, in the case of Thailand, this option is cost-effective only at $10/tCO_2_eq and higher carbon price scenarios.

### Mitigation/sequestration from LULUCF sector

**Fig. 9:**
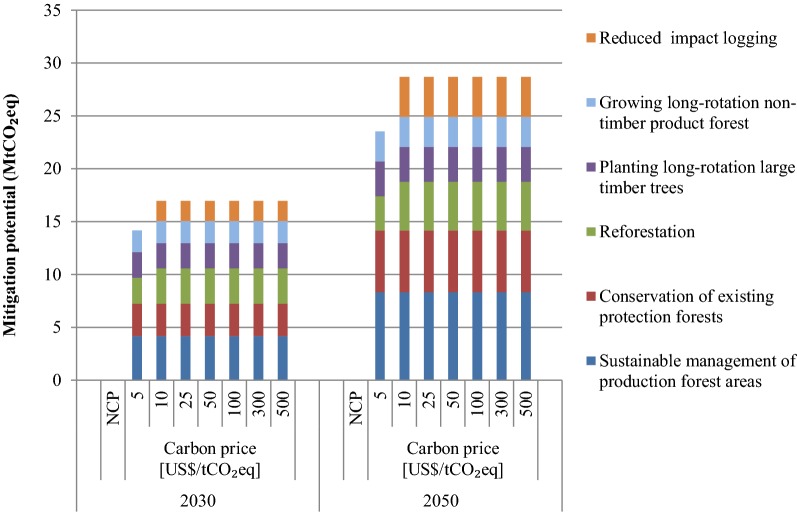
GHG sequestration potential in the LULUCF sector in 2030 and 2050 in various scenarios

This study finds that LULUCF does not have any no-regret mitigation measure i.e., there are no mitigation options at negative net cost. Several options, such as sustainable management of production forest areas, conservation of existing protection forests and reforestation, planting long-rotation large timber trees, and growing long-rotation non-timber product forest are cost-effective options at $5/tCO_2_eq in the forestry sector and the mitigation/sequestration potential would be 14.2 MtCO_2_eq in 2030 (see Fig. 9). At $10/tCO_2_eq, in addition to the options in the $5/tCO_2_eq scenario, reduced impact logging would also be cost effective. At $25/tCO_2_eq and higher carbon price scenarios, the mitigation potential would be the same as that in $10/tCO_2_eq scenario. The mitigation/sequestration potential at $10 and higher would be 17 MtCO_2_eq in 2030.

The mitigation/sequestration options in all scenarios in 2050 would be the same as in 2030. At $5 and $10 per tCO_2_eq, the mitigation/sequestration potential would be 23.6 and 28.7 MtCO_2_eq. The mitigation potential in 2050 in other higher carbon price scenarios would be the same as in $10 per tCO_2_eq scenario, i.e., 28.7 MtCO_2_eq.

### Mitigation and sequestration in the AFOLU

**Fig. 10:**
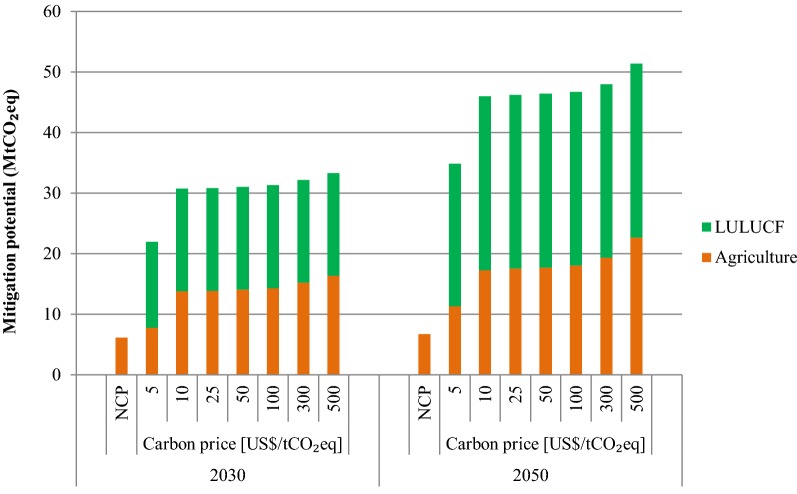
GHG mitigation/sequestration potential in various scenarios in the AFOLU sector

The mitigation/sequestration potential from the AFOLU as a whole in 2030 and 2050 in both the NCP and carbon price scenarios is presented in Fig. 10. In 2030, the mitigation potential would be 6.1 MtCO_2_eq in the NCP scenario. In the same year, the mitigation/sequestration potential would increase to 22.0 MtCO_2_eq at $5/tCO_2_eq and 30.8 MtCO_2_eq at $10/tCO_2_eq. At higher carbon price rates, there is no significant increase in the potential. The mitigation/sequestration potential would be 33.3 MtCO_2_eq at $500 per tCO_2_eq, respectively.

In 2050, in the NCP scenario, the mitigation potential would be 6.8 MtCO_2_eq. At $5, $10 and $25 per tCO_2_eq, mitigation/sequestration potential would be 34.9, 46.0 and 46.2 MtCO_2_eq, respectively. At higher emission scenarios, i.e. $50, $300 and $500 per tCO_2_eq, the mitigation/sequestration potential would be 46.4, 48.0 and 51.4 MtCO_2_eq, respectively.

It should be noted that at relatively lower carbon price of $10/tCO_2_eq, a significant amount of mitigation/sequestration will be achievable. In higher carbon price scenarios, the additional mitigation/sequestration potential that would be achievable is relatively insignificant. In agriculture sector, at very high carbon price of $500/tCO_2_eq, additional mitigation potential exists. Higher proportion of mitigation potential is achievable at lower carbon prices. Smith et al. [1] stated in the Fifth Assessment report of the IPCC that the agricultural mitigation in cropland and grazing land management are achievable at lower carbon prices up to $20 per tCO_2_eq. It should be noted that mitigation potential are sensitive to countermeasure costs. There are wide ranges of countermeasure costs in the literature and country specific data are not available. Also there lies high uncertainty in the emissions as well as mitigation potential in the AFOLU sector.

In the case of forestry, studies show that the cost of mitigation measures in developing countries are in the range of $0.5 to $7 per tCO_2_, while for industrialized nation the cost is in the range of $1.4 to $22 per tCO_2_. Graham et al. [14] reported that the cost of reducing emissions in Southeast Asia is in the range of $2.5 to $20.5 per tCO_2_eq removed. Therefore, increasing carbon price in forestry above $25 per tCO_2_eq would not have any changes in the mitigation potential as the countermeasure options considered in the study have lower abatement cost (i.e. less than $25 per tCO_2_eq). Similar findings were observed in the analysis of potential and costs of LULUCF use by European Union member countries [37]. An increase in the marginal cost above EUR 50 per tCO_2_ would not induce significant additional removals of CO_2_. The sensitivity analyses in this study show that increasing the countermeasure costs by 100% would not change the total mitigation potential from forestry sector but the carbon price needed to achieve the same sequestration would be higher ($50 per tCO_2_eq). It should also be noted that the cost and mitigation potential data in LULUCF has very high uncertainty [5, 38].

### Net emission/sequestration in various scenarios

Table 5 presents the emissions/sequestration in the agriculture and LULUCF sectors, as well as in the overall AFOLU sector. In 2030, total emissions from AFOLU in NCP will be half of the BAU level. At $5/tCO_2_eq and higher carbon price scenarios, total emissions will be negative, i.e. there will be overall sequestration from AFOLU sector due to higher sink capacity than the emission sources.

**Table 5:** Emissions/sequestration in 2030 and 2050 from Agriculture, LULUCF and AFOLU

Year	Emissions	BAU	NCP	Carbon price $/tCO_2_eq						
				5	10	25	50	100	300	500
2030	Agriculture	51.2	45.1	43.4	37.4	37.3	37.1	36.9	36.0	34.8
	LULUCF	− 39.0	− 39.0	− 53.2	− 56.0	− 56.0	− 56.0	− 56.0	− 56.0	− 56.0
	Total AFOLU	12.2	6.1	− 9.8	− 18.6	− 18.6	− 18.8	− 19.1	− 20.0	− 21.1
2050	Agriculture	63.6	56.8	52.3	46.3	46.1	45.9	45.6	44.3	40.9
	LULUCF	− 39.0	− 39.0	− 62.6	− 67.7	− 67.7	− 67.7	− 67.7	− 67.7	− 67.7
	Total AFOLU	24.6	17.8	− 10.3	− 21.4	− 21.6	− 21.8	− 22.1	− 23.4	− 26.8

− represents net sequestration

Similarly, in 2050, there will be net emissions in the BAU and NCP scenarios, whereas in all the carbon price scenarios, there will be net sequestration from the AFOLU sector. Net sequestration will be in the range of 10.3 MtCO_2_eq at $5/tCO_2_eq and 26.8 MtCO_2_eq at $500/tCO_2_eq.

### Mitigation/sequestration in AFOLU at $10/tCO_2_eq

**Fig. 11:**
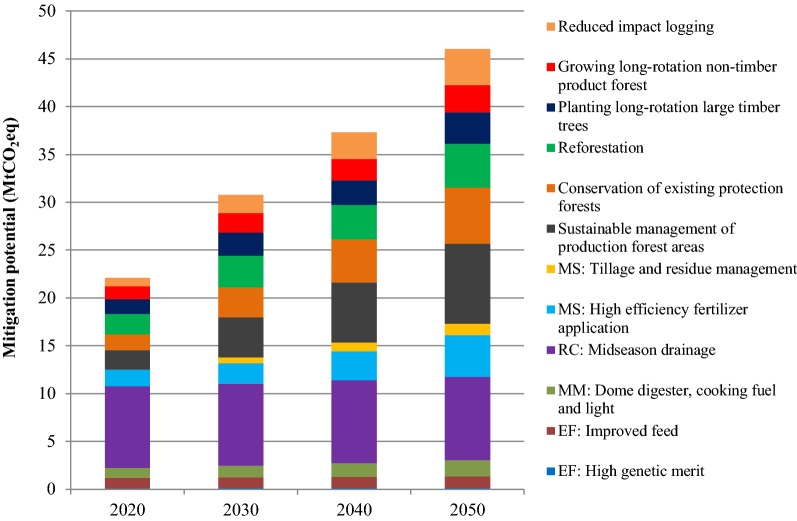
GHG mitigation potential in the AFOLU sector at carbon price of $10/tCO_2_eq during 2020–2050

At a carbon price of $10/tCO_2_eq, GHG mitigation potential from the AFOLU sector during 2020–2050 is shown in Fig. 11. At this carbon price, the emission reduction (or mitigation) potential in the agriculture sector would be 12.5 MtCO_2_eq in 2020 and would increase to 17.3 MtCO_2_eq in 2050. The mitigation potential in 2020 would be 26.8% of the total agriculture emission in the BAU, whereas the mitigation potential will increase slightly to 27.2% in 2050. Out of total mitigation potential in 2020, midseason drainage in rice cultivation would account for 68.1% of the reduction. Tillage and residue management and high efficiency fertilizer application combined in managed soils will account for 14% of the reduction in the same year. In enteric fermentation, improved feed and high genetic merit would mitigate 8.8% and 0.9% of the total emission from agriculture, respectively. Dome digesters in manure management will amount to 8.1% of the total reduction potential.

In 2050, the share of mitigation from rice cultivation by mid-season drainage will reduce to 50.2% of the total potential in agriculture. In managed soils, tillage and residue management and high efficiency fertilizer application will contribute 7.0% and 25.3%, respectively. Dome digesters in manure management will be 9.8% of the total mitigation potential. Likewise, improved feed and high genetic merit will reduce the emission from enteric fermentation process, which will be 7.0% and 0.8% of the total mitigation potential.

In LULUCF, the additional sequestration potential through mitigation measures in 2020 will be 9.6 MtCO_2_eq, while it will increase to 28.7 MtCO_2_eq in 2050. In 2050, sustainable management of production forest areas will contribute to the highest sequestration potential in the sector, followed by conservation of existing protection forests, reforestation, planting long-rotation large timber trees, reduced impact logging, and growing long-rotation non-timber product trees.

## Conclusions

The AFOLU sector produces one-fourth of the global GHG emissions. There is no policy in Thailand addressing GHG mitigation in the agriculture sector. However, in the case of the land-use sector, Thailand’s NDC has stated that the country has a target to maintain 40% of total land area as forest. The forest area in 2015 was only about 32%. This study quantified the GHG emissions from the AFOLU sector in a BAU scenario during 2015–2050. In addition, the study has identified potential mitigation options in the agriculture sector and mitigation as well as sequestration options in the LULUCF sector, without carbon price in the NCP scenario and with carbon price scenarios.

GHG emission in the BAU scenario would increase from 45.3 MtCO_2_eq in 2015 to 63.6 MtCO_2_eq in 2050. Rice cultivation is the major source of GHG emission in the agriculture sector in 2015, and it would remain the major source of GHG emission during 2015 to 2050. Emission from managed soils, which is the second highest (21.6% in 2015) contributor to agriculture related GHG emission, would have a share of 37.1% by 2050. The share of emission from enteric fermentation in ruminant animals would decrease from 13.2% in 2015 to 10.1% in 2050. Manure management, which accounts for 9.1% of emissions from the agriculture sector, would have a share of 11.9% in 2050.

Net sequestration from LULUCF would increase from 37 MtCO_2_eq in 2015 to 39 MtCO_2_eq in 2050. Forest and grassland conversion as well as emissions from soil are the sources of emissions from the LULUCF sector, whereas changes in forest and other biomass woody stocks are the source of sequestration.

The NCP scenario shows that improved feed to livestock, dome digesters in manure management and incorporation of off-season straw in rice cultivation are optimal solutions even without the carbon price. At $5/tCO_2_eq, high efficiency fertilizer application would become an optimal solution. At $10/tCO_2_eq and $25/tCO_2_eq, high genetic merit and improved feed in enteric fermentation, dome digesters in manure management, mid-season drainage in rice cultivation, and high efficiency fertilizer application as well as tillage and residue management in managed soils are the optimal countermeasure solutions. At $50, $100 and $300, the daily spread of manure in manure management in addition to the combination of countermeasures at $10 and 25/tCO_2_eq are the effective countermeasure options. At $500/tCO_2_eq, application of slow-release fertilizer in addition to the countermeasures in prior scenarios is the cost-effective option.

In 2030, the NCP scenario would have mitigation potential of 6.1 MtCO_2_eq from the agriculture sector. Similarly, in the carbon price scenarios, mitigation potential in the agriculture sector at $5, $10, $25 and $50 per tCO_2_eq would be 7.8, 13.8, 13.9 and 14.1 MtCO_2_eq, respectively. At $100, $300 and $500 per tCO_2_eq, mitigation potential would be 14.3, 15.2 and 16.4 MtCO_2_eq, respectively. Likewise, in 2050, mitigation potential from the agriculture sector in the NCP scenario would be 6.8MtCO_2_eq. In the carbon price scenario, the mitigation potential would be in the range between 11.3 MtCO_2_eq at $5/tCO_2_eq and 22.7 MtCO_2_eq at $500/tCO_2_eq.

In the BAU scenario, net sequestration or sink capacity in the LULUCF sector would be in the range of 37.0 to 40.0 MtCO_2_eq during 2015–2050. Some of the countermeasures that would achieve additional sequestration in the LULUCF sector include conservation of existing protection forests, reforestation, planting timber trees, planting oil palm, reduced impact logging, and sustainable management of production forests. In the NCP scenario, no countermeasure is found to be a no-regret option. In 2030, sequestration potential at $5 and $10 per tCO_2_eq would be 14.2 MtCO_2_eq and 17.0 MtCO_2_eq, respectively. At $25 per tCO_2_eq and higher carbon prices, sequestration potential would be the same as in $10 per tCO_2_eq i.e., 17.0 MtCO_2_eq. At $5/tCO_2_eq, cost-effective measures include sustainable management of production forest areas, conservation of existing protection forests and reforestation, planting long-rotation large timber trees, and growing long-rotation non-timber product forest. At $10/tCO_2_eq, reduced impact loading would also become a cost-effective measure in addition to other measures in prior scenarios. In 2050, sequestration potential would be 23.6 MtCO_2_eq at $5/tCO_2_eq and 28.7 tCO_2_eq at $10/tCO_2_eq and higher carbon prices.

Although this study has quantified GHG mitigation potential from the AFOLU sector at various carbon prices, there are socio-economic, technological, and ecological as well as institutional barriers and other challenges for the implementation of these measures [1]. In addition, the monitoring and verification of the emissions related to LULUCF sector is also a barrier to further reduction. Other issues that are associated with additionality, leakage and permanence have led to controversies and are the critical barriers [5, 38]. Proper land and livestock management practices and efficient policies for mitigation are needed. Integrating co-benefits of climate change policies, improvement in environmental quality, sustainable development, and food and energy security can make it more attractive to policy makers as well as to farmers and land owners. One of the most important factors will be the financial incentives. Attractive financial incentives are important to implement mitigation strategies. Modularity, like the Joint Implementation and Clean Development Mechanism, can also be effective in the implementation of mitigation projects [39].

Thailand’s forest coverage (in terms of percentage of total land) is quite low when compared to some industrialized nation in Asia and Europe such as Japan, Korea, Sweden and Finland. The maximum potential of forest area in Thailand has not been assessed yet. Forest has important role in carbon sequestration. This study has assumed the forest coverage to reach 40% and remain constant. Further studies would be needed to assess the full potential of forest coverage and its corresponding mitigation.

## Highlights

- The study estimated greenhouse gases emissions from AFOLU sector in the Business as usual (BAU) scenario during 2015–2050 and analyzed the mitigation/sequestration potential at different carbon prices.
- No-regret mitigation options has been identified.
- The study used AFOLU-B, which is a bottom-up model, for the analysis.
- Net sequestration in AFOLU sector will be possible with mitigation/sequestration measures.

## Abbreviations

AFOLU: Agriculture, Forestry and Other Land Use
AFOLUB: Agriculture, Forestry and Other Land Use Bottom-up
AG: agriculture
AIM/CGE: Asia-pacific Integrated Model/Computable General Equilibrium
BAU: business-as-usual
CAER: Center for Applied Economic Research
CAGR: compound annual growth rate
CH_4_: methane
CO_2_: carbon dioxide
CO_2_eq: carbon dioxide equivalent
EF: enteric fermentation
EUR: Euro
GHG: greenhouse gas
ha: hectare
INDC: Intended Nationally Determined Contributions
IPCC: Intergovernmental Panel on Climate Change
LU: land use
LULUCF: Land Use, Land Use Change and Forestry
MAC: marginal abatement costs
MM: manure management
MS: managed soils
NCP: No Climate Policy
NDC: Nationally Determined Contributions
NESDB: Office of the National Economic and Social Development Board
NIES: National Institute for Environmental Studies
N_2_O: nitrous oxide
OAE: Office of Agricultural Economics
ONEP: Office of Natural Resources and Environmental Policy and Planning
O&M: operations and maintenance
RC: rice cultivation
REDD+: Reducing Emissions from Deforestation and forest Degradation plus
UNFCCC: United Nations Framework Convention on Climate Change
US$: United States dollar
USEPA: United States Environmental Protection Agency
yr: year

## References

[1] Smith P, Bustamante M, Ahammad H, Clark H, Dong H, Elsiddig EA, Haberl H, Harper R, House J, Jafari M, Masera O, Mbow C, Ravindranath NH, Rice CW, Abad CR, Romanovskaya A, Sperling F, Tubiello FN, Edenhofer O, Pichs-Madruga R, Sokona Y (2014). Agriculture, Forestry and Other Land Use (AFOLU). Climate change 2014: mitigation of climate change. Contribution of working group III to the fifth assessment report of the intergovernmental panel on climate change.

[2] Office of Natural Resources and Environmental Policy and Planning (ONEP) (2010). Thailand’s Second National Communication under the United Nations Framework convention on climate change.

[3] Office of Natural Resources and Environmental Policy and Planning (ONEP) (2015). Thailand’s Intended Nationally Determined Contribution (INDC).

[4] Federici S, Tubiello FN, Salvatore M, Jacobs H, Schmidhuber J (2015). New estimates of CO_2_ forest emissions and removals: 1990–2015. For Ecol Manage.

[5] Grassi G, House J, Dentener F, Federici S, den Elzen M, Penman J (2017). The key role of forests in meeting climate targets requires science for credible mitigation. Nat Climate Change.

[6] Food and Agriculture Organization of the United Nations (FAO) (2015). Global Forest Resources Assessment 2015: Desk reference.

[7] World Development Indicators. Forest Area. (2018) The World Bank Group. https://data.worldbank.org/indicator/AG.LND.FRST.ZS. Accessed 25 Sept 2018.

[8] United States Environmental Protection Agency (USEPA) (2013). Global mitigation of Non-CO_2_ greenhouse gases: 2010–2030.

[9] Graus W, Harmelink M, Hendriks C (2004). Marginal GHG-abatement curves for agriculture.

[10] Intergovernmental Panel on Climate Change (IPCC) (ed) (2014) Climate change 2014: mitigation of climate change. Contribution of working group III to the fifth assessment. Report of the intergovernmental panel on climate change. Cambridge University Press, Cambridge.

[11] Nabuurs GJ, Masera O, Andrasko K, Benitez-Ponce P, Boer R, Dutschke M, Elsiddig E, Ford-Robertson J, Frumhoff P, Karjalainen T, Krankina O, Kurz WA, Matsumoto M, Oyhantcabal W, Sanchez MJS, Zhang X, Metz B, Davidson O, Bosch P, Dave R, Meyer L (2007). Forestry. Climate change 2007: mitigation. Contribution of working group III to the fourth assessment report of the intergovernmental panel on climate change.

[12] Cacho OJ, Hean RL, Wise RM (2003). Carbon-accounting methods and reforestation incentives. Aust J Agric Resour Econ.

[13] Richards KR, Stokes C (2004). A review of forest carbon sequestration cost studies: a dozen years of research. Climatic Change.

[14] Graham V, Laurance SG, Grech A, McGregor A, Venter O (2016). A comparative assessment of the financial costs and carbon benefits of REDD+ strategies in Southeast Asia. Environ Res Lett.

[15] Center for Applied Economic Research (CAER) (2018). Support to the development and implementation of the Thai climate change policy.

[16] Jilani T, Hasegawa T, Matsuoka Y (2015). The future role of agriculture and land use change for climate change mitigation in Bangladesh. Mitig Adapt Strat Glob Change.

[17] Pradhan BB, Shrestha RM, Hoa NT, Matsuoka Y (2017). Carbon prices and greenhouse gases abatement from agriculture, forestry and land use in Nepal. Glob Environ Change.

[18] Hoa NT, Hasegawa T, Matsuoka Y (2014). Climate change mitigation strategies in agriculture, forestry and other land use sectors in Vietnam. Mitig Adapt Strat Glob Change.

[19] Hasegawa T, Matsuoka Y (2012). Greenhouse gas emissions and mitigation potentials in agriculture, forestry and other land use in Southeast Asia. J Integr Environ Sci.

[20] Hasegawa T, Matsuoka Y (2015). Climate change mitigation strategies in agriculture and land use in Indonesia. Mitig Adapt Strat Glob Change.

[21] Hasegawa T, Fujimori S, Masui T, Matsuoka Y (2016). Introducing detailed land-based mitigation measures into a computable general equilibrium model. J Clean Prod.

[22] Food and Agriculture Organization of the United Nations (FAO) (2009). Thailand forestry outlook study (trans: Pacific ROfAat).

[23] Land Resource: Land Use data in Thailand. Land Development Department. 2016. http://www1.ldd.go.th/ldd/. Accessed 15 June 2018.

[24] Office of Natural Resources and Environmental Policy and Planning (ONEP) (2010). Thailand’s Second National Communication under the United Nations Framework Convention on Climate Change.

[25] Office of Natural Resources and Environmental Policy and Planning (ONEP) (2018). Thailand’s Third National Communication.

[26] Office of Agricultural Economics (2008). Agricultural Statistics of Thailand 2007 (trans: Ministry of Agriculture and Cooperatives).

[27] Office of Agricultural Economics (2011). Agricultural Statistics of Thailand 2010 (trans: Ministry of Agriculture and Cooperatives).

[28] Office of Agricultural Economics (2014). Agricultural Statistics of Thailand 2013 (trans: Ministry of Agriculture and Cooperatives).

[29] Office of Agricultural Economics (2016). Agricultural Statistics of Thailand 2015 (trans: Ministry of Agriculture and Cooperatives).

[30] Office of the National Economic and Social Development Board (NESDB) (2013). Population projections for Thailand 2010–2040.

[31] Intergovernmental Panel on Climate Change (IPCC) (2008). 2006 IPCC Guidelines for National Greenhouse Gas Inventories—a primer.

[32] Bates J, Brophy N, Harfoot M, Webb J (2009) Agriculture: methane and nitrous oxide. Sectoral emission reduction potentials and economic costs for climate change.

[33] Bates J, Schneider T (1998). A strategy for reducing methane emissions. Studies in environmental science.

[34] AEA Technology Environment (AEATE) (1998) Options to reduce methane emissions (Final Report).

[35] United States Environmental Protection Agency (USEPA) (2006). Global mitigation of non-CO_2_ greenhouse gases.

[36] Smith P, Martino D, Cai Z, Gwary D, Janzen H, Kumar P, McCarl B, Ogle S, O’Mara F, Rice C, Scholes B, Sirotenko O, Howden M, McAllister T, Pan G, Romanenkov V, Schneider U, Towprayoon S, Wattenbach M, Smith J (2008). Greenhouse gas mitigation in agriculture. Philos Trans R Soc B Biol Sci.

[37] Böttcher H, Verkerk H, Gusti M, Havlík P (2011) Analysis of potential and costs of LULUCF use by EU Member States.

[38] Siikamaki J, Ferris J, Munnings C (2012) Land Use, Land-Use Change, and Forestry Offsets Resources for the future. Washington DC.

[39] Verchot LV (2014). Challenges and opportunities for mitigation in the agriculture sector.

